# Supplementary material for: Detailed analysis of chick optic fissure closure reveals Netrin-1 as an essential mediator of epithelial fusion
Source: eLife. 2019 Jun 4;8:e43877. doi: 10.7554/eLife.43877 (PMC6606025; doi:10.7554/eLife.43877)
Supplement: Transparent reporting form [file elife-43877-transrepform.docx]

***eLife’s* transparent reporting form**

We encourage authors to provide detailed information *within their submission* to facilitate the interpretation and replication of experiments. Authors can upload supporting documentation to indicate the use of appropriate reporting guidelines for health-related research (see [EQUATOR Network](http://www.equator-network.org/%20)), life science research (see the [BioSharing Information Resource](https://biosharing.org/)), or the [ARRIVE guidelines](http://www.plosbiology.org/article/info:doi/10.1371/journal.pbio.1000412) for reporting work involving animal research. Where applicable, authors should refer to any relevant reporting standards documents in this form.

If you have any questions, please consult our Journal Policies and/or contact us: [editorial@elifesciences.org](mailto:editorial@elifesciences.org).

**Sample-size estimation**

- You should state whether an appropriate sample size was computed when the study was being designed
- You should state the statistical method of sample size computation and any required assumptions
- If no explicit power analysis was used, you should describe how you decided what sample (replicate) size (number) to use

**For phenotypic analysis** of *Netrin-1* deficient mice and zebrafish, the primary testable outcome was the presence or absence of coloboma in the ventral retina – a dichotomous variable. The *Null Hypothesis* was that Netrin-1 null embryos will display no ocular phenotypic difference to wild types within the same cross. Sample size calculations for the phenotyping experiments were performed using methods outlined by Dell^1^ for dichotomous outcome data comparing two groups. On this basis, taking alpha at 0.05, to have 80% power to detect the presence of an eye malformation (*d*=1; penetrance = 100%), 4 embryos per group (WT versus *Netrin-1* null) would be required to reject the null hypothesis. For 50% penetrance, n = 14. No coloboma was observed in wild-type or heterozygous animals in either species.

Reference: 1) Dell RB Ramakrishnan R. *Ilar J* **43,** 207–213 (2002).

Please outline where this information can be found within the submission (e.g., sections or figure legends), or explain why this information doesn’t apply to your submission:

**Replicates**

- You should report how often each experiment was performed
- You should include a definition of biological versus technical replication
- The data obtained should be provided and sufficient information should be provided to indicate the number of independent biological and/or technical replicates
- If you encountered any outliers, you should describe how these were handled
- Criteria for exclusion/inclusion of data should be clearly stated
- High-throughput sequence data should be uploaded before submission, with a private link for reviewers provided (these are available from both GEO and ArrayExpress)

Please outline where this information can be found within the submission (e.g., sections or figure legends), or explain why this information doesn’t apply to your submission:

**For chick staging** and fusion progression data, one eye per chick embryo was used in all analyses throughout this study. Cognate eyes from the same embryo were never used.

**For RNASeq anaylsis**, each sample (e.g. fissure, whole-eye, dorsal or ventral eye) was a collection of pooled tissue taken from >10 separate eyes. One pool per sample type was used per RNAseq IonProton chip run. This was considered one biological replicate. Data was taken from each of the three independently collected biological replicate and independently run as technical replicates. To identify the relationships between samples, Log2 transformed counts per million were calculated using edgeR and Spearman’s rank correlation was used to identify the similarities in genome-wide expression levels between samples. Genes not expressed in at least three samples were excluded. One outlier was encountered throughout all 30 RNAseq samples (Fiss_E5-3) but was retained in the analysis. Figure S3 clearly shows Pearson’s correlation coefficient and outlier data for all RNAseq data used. All RNAseq data files are submitted to the NCBI Gene Expression Ominibus database (<http://www.ncbi.nlm.nih.gov/geo>) with the accession number GSE84916.

In Figure 3 we present mean TPM values where n=3 for genes involved in biological adhesion revealed by enrichment analysis. Standard deviations are provided as error bars. The figure legend reads: “Analysis of normalised mean expression values (TPM, n=3 technical replicates; error bars = 1x standard deviation)”.

URL details for Reviewer access to raw RNAseq data:

<http://www.ncbi.nlm.nih.gov/geo/query/acc.cgi?token=opgraigsffezhqb&acc=GSE84916>

**For qualitative and quantitative** **analyses between tissue samples** (e.g. mouse, chick and human immunofluorescence assays), a minimum of three (*n* ≥3) samples were used, with each sample prepared on separate occasions (i.e. 3x technical replicates). For analysis, samples were anonymized and/or analyzed by a separate researcher.

In Figure 1, we present mean numbers of Phospho-Histone H3 immunofluorescence data from confocal analyses of flat mounted fissures. The figure legend states n values, error bars are 1x standard deviations, and that p-values (shown in figure) are calculated using a paired *t*-test.

For Basement membrane remodeling and epithelial characteristic changes (Figure 2a-b), the accompanying figure legend states: “Images are from a single OFM and are representative of n=3 samples”.

In Figure 2d, Activated Caspase immunofluorescence data are provided as the mean of 4x biological replicates processed as 2x technical replicates. Error bars are 95% confidence intervals and figure legend text reads: “Data in histograms are presented for the mean of all measurements; *n* =4; error bars = 95% confidence intervals”.

**Statistical reporting**

- Statistical analysis methods should be described and justified
- Raw data should be presented in figures whenever informative to do so (typically when N per group is less than 10)
- For each experiment, you should identify the statistical tests used, exact values of N, definitions of center, methods of multiple test correction, and dispersion and precision measures (e.g., mean, median, SD, SEM, confidence intervals; and, for the major substantive results, a measure of effect size (e.g., Pearson's r, Cohen's d)
- Report exact p-values wherever possible alongside the summary statistics and 95% confidence intervals. These should be reported for all key questions and not only when the p-value is less than 0.05.

Please outline where this information can be found within the submission (e.g., sections or figure legends), or explain why this information doesn’t apply to your submission:

N numbers per test, error bars, p-values and any statistical tests used for comparative analysis are embedded in figure legends and in relevant sections of Methods section.

**For RNAseq**, quantitative analyses applied a false discovery rate (FDR) adjusted *p*-value < 0.05, as described in materials and methods section of the main manuscript.

**For enrichment-based ontology** searches, GOrilla generated *p* values were used (Figure S3; see Eden et al., <https://doi.org/10.1186/1471-2105-10-48)>.

(For large datasets, or papers with a very large number of statistical tests, you may upload a single table file with tests, Ns, etc., with reference to sections in the manuscript.)

**Group allocation**

- Indicate how samples were allocated into experimental groups (in the case of clinical studies, please specify allocation to treatment method); if randomization was used, please also state if restricted randomization was applied
- Indicate if masking was used during group allocation, data collection and/or data analysis

Please outline where this information can be found within the submission (e.g., sections or figure legends), or explain why this information doesn’t apply to your submission:

For phenotypic analysis of *Netrin-1 deficient* mice and zebrafish, the primary testable outcome was the presence or absence of coloboma in the ventral retina – a dichotomous variable. Raw phenotyping analysis was performed without knowledge of genotype data. Genotyping was then matched to phenotype data *post hoc.*

Samples for RNAseq were grouped based on tissue regions dissected, as described in Methods section.

**Additional data files (“source data”)**

- We encourage you to upload relevant additional data files, such as numerical data that are represented as a graph in a figure, or as a summary table
- Where provided, these should be in the most useful format, and they can be uploaded as “Source data” files linked to a main figure or table
- Include model definition files including the full list of parameters used
- Include code used for data analysis (e.g., R, MatLab)
- Avoid stating that data files are “available upon request”

Please indicate the figures or tables for which source data files have been provided:

Source data files are provided for RNAseq analysis:

**Source File 1** – Kallisto analysis of RNAseq data from segmentally dissected HH.St25-26/E5 chick eyes.

**Source File 2** - Limma analysis of RNAseq data from segmentally dissected chick eyes at all stages
